# Precocial parasympathetic-mediated cardiac regulation in embryonic gulf killifish (*Fundulus grandis*)

**DOI:** 10.1242/jeb.251772

**Published:** 2026-04-30

**Authors:** Steven L. Williams, Benjamin Dubansky, Jack Eudy, Brandon Chow, Katelin Ratcliff, Warren W. Burggren

**Affiliations:** ^1^ Developmental Integrative Biology Research Group, Department of Biological Sciences, University of North Texas, Denton, TX 76203, USA; ^2^ Department of Biological and Agricultural Engineering, Louisiana State University, Baton Rouge, LA 70803, USA; ^3^ Department of Chemistry, University of New Orleans, New Orleans, LA 70148, USA; ^4^ Developmental, Inc., Baton Rouge, LA 70810, USA

**Keywords:** Vagal tone, Autonomic control, Heart, Embryo, Startle, Development

## Abstract

Embryonic fishes provide valuable models for understanding the ontogeny of vertebrate cardiovascular regulation, yet the timing of autonomic control onset varies widely among species. In most teleosts, it is generally accepted that parasympathetic (vagal) influence over heart rate arises only near hatching, but historical observations suggest that killifishes deviate from this paradigm. Here, we characterized the development of vagal control in Gulf killifish (*Fundulus grandis*), a precocial species with an extended embryonic period. Using both direct mechanical and indirect vibrational startle stimuli, combined with atropine treatment, we identified two distinct cardiac response phenotypes: (1) a transient atrial and ventricular arrest evident by 70% of embryonic development, and (2) a later-developing transient bradycardia beginning at 80%. Both responses were abolished by atropine, confirming parasympathetic mediation, and neither habituated to repeated stimulation. These findings demonstrate that *F. grandis* embryos acquire functional vagal tone well before hatching, contrasting with the delayed autonomic development typical of most vertebrates. Thus, we expand upon early observations from nearly a century-old study, establishing the Gulf killifish as a tractable model for investigating the developmental physiology of early autonomic control in vertebrates.

## Teaser

**Summary:** Embryonic Gulf killifish exhibit atropine-sensitive bradycardia and startle-induced cardiac responses well before hatching, demonstrating that functional parasympathetic regulation of heart rate develops precocially in this species.

## INTRODUCTION

The onset of cardioregulatory control in developing vertebrates generally occurs around hatching or birth, marking the emergence of functional autonomic regulation of the heart. This developmental paradigm is broadly conserved across taxa, though exceptions do occur at the species level (Joyce and Wang, 2022; Taylor et al., 2014). In teleost (bony) fishes, most studies report little deviation from this model. However, given the considerable diversity among the ∼30,000 known teleost species occupying a wide range of aquatic habitats, the developmental emergence of autonomic control, particularly parasympathetic cardiac regulation, likely varies among species.

Developmental trajectories across vertebrates, particularly in birds and teleost fishes, are often categorized as precocial or altricial based on the functional maturity of key phenotypic traits at hatching (Dubansky, 2018). Precocial species hatch with relatively mature and functional organ systems, whereas altricial species are comparatively immature and instead undergo substantial functional development post-hatch (Ricklefs and Starck, 1998). More accurately, this distinction is best viewed as a continuum of developmental states rather than a strict dichotomy, with species differing in their degree of maturity at hatch (Augustine et al., 2019). While environmental factors such as temperature can alter species-specific times to hatch, they exert little influence on whether species follow their established precocial or altricial developmental trajectories (Ricklefs and Starck, 1998).

In the altricial zebrafish (*Danio rerio*), for example, after hatching at ∼3 days post-fertilization (dpf), the earliest sign of vagal regulation of heart rate (*f*_H_) only appears at 5 dpf (Mann et al., 2010; Schwerte et al., 2006). A similar post-hatch onset of autonomic control has also been reported in medaka (*Oryzias latipes*), rainbow trout (*Oncorhynchus mykiss*) and the golden mullet (*Liza aurata*) (Balashov et al., 1991; Miller et al., 2011; Watanabe-Asaka et al., 2022). Notably, even when cholinergic or adrenergic receptors are pharmacologically functional at earlier stages, there is little evidence of centrally mediated autonomic regulation of *f*_H_ (Miller et al., 2011).

In contrast, both the Atlantic killifish (*Fundulus heteroclitus*) and Gulf killifish (*Fundulus grandis*) are precocial species, possessing a comparatively advanced physiological maturity at hatch. The incubation period for *Fundulus* embryos can range from 10 to 16 days, depending on species, temperature and other environmental variables (Armstrong and Child, 1965; Dubansky et al., 2013). This duration is substantially longer than the ∼72 h incubation period of zebrafish at 28°C or the ∼48 h period of golden mullet at 20°C (Balashov et al., 1991; Mann et al., 2010). Yet, despite variation in times to hatch among killifish (often spanning several days), the developmental maturity of functional traits such as autonomic regulation of *f*_H_ remains consistent within both species. *Fundulus* species are therefore a well-suited model for investigating the ontogeny of autonomically mediated cardiac regulation in precocial teleosts, owing to their extended embryonic development and experimental accessibility.

Nearly a century ago, Armstrong (1931) reported an unusually early onset of parasympathetic control of cardiac rhythm in Atlantic killifish embryos. When lightly pressed under a flexible mica coverslip, 9 dpf embryos (approximately 60–70% of development at 20°C) exhibited a transient cardiac arrest in response to mechanical stimulation, which Armstrong attributed to vagal inhibition. At the time, the ontogeny of the autonomic nervous system was poorly understood, making these observations an exception to the prevailing vertebrate paradigm of the ontogeny of autonomic nervous system efficacy (Taylor et al., 2014).

The present study revisits and expands upon Armstrong's (1931) original observations by characterizing the ontogeny of parasympathetic cardiac regulation in the embryonic Gulf killifish, a sister species of the Atlantic killifish. Specifically, we sought to (i) determine the developmental window for the onset of vagal innervation with respect to alterations in *f*_H_, (ii) identify distinct startle-induced cardiac response phenotypes elicited by different methods of vagal stimulation, (iii) assess whether these responses habituate with repeated stimuli, and (iv) investigate the mechanistic basis underlying sinus venosus, atrial and ventricular responses to vagal stimulation.

## MATERIALS AND METHODS

### Animal husbandry and embryo rearing

Embryos from two separate breeding populations of laboratory-reared, wild type adult Gulf killifish, *Fundulus grandis* Baird & Girard 1853, were obtained from Louisiana State University (Baton Rouge, LA, USA) and Baylor University (Waco, TX, USA). Eggs were either express-mailed or personally transported under controlled conditions to the University of North Texas (Denton, TX, USA), where all experimentation was performed. During the studied embryonic stages, sex could not be determined. Each developmental stage was represented by an equal number of embryos from each batch collected on consecutive days to mitigate batch effects and reduce inter-clutch variability. All procedures involving embryonic Gulf killifish were conducted in accordance with institutional guidelines. Experiments used embryonic stages only and were therefore exempt from IACUC review.

Adults were maintained in recirculating saltwater systems at 24°C, pH 7.8 and 10–12 ppt salinity (17–20 mS cm^−1^ conductivity) under a 14 h:10 h light:dark cycle. Aquaxcel 0.8 mm pellet feed (Cargill^®^, Wayzata, MN, USA) was provided once daily on non-breeding days and twice daily prior to spawning. Water chemistry was monitored weekly to monitor nitrogen and pH stability. Egg-collection mats were placed the evening before breeding and retrieved 1 h post-spawn to allow sufficient time for fertilization.

Embryos were reared in Petri dishes atop moistened filter paper, wetted twice daily with isotonic (10–12 ppt) artificial seawater (ASW; Instant Ocean^®^, Spectrum Brands, Blacksburg, VA, USA) and incubated continuously at 22°C.

Developmental progress was expressed as percentage of embryonic development rather than days post-fertilization to account for variation in hatching time, which ranges from 10 to 14 dpf at 20–22°C (Dubansky et al., 2013). The 12 dpf time point was designated as the hatching endpoint (100%) because embryos at this stage most frequently hatched spontaneously at 22°C.

### Measuring embryonic *f*_H_ and sinus venosus beat frequency

Two startle-stimulus assays were used: a direct physical stimulus involving gentle compression of the embryos and an indirect vibrational stimulus. Because applying each form of startle stimulus involved distinct techniques, separate methods were required to measure *f*_H_ rate. *f*_H_ is defined as the frequency of coupled atrial and ventricular beats; individual chamber beat frequencies [sinus venosus (SV), atrium, ventricle] were also monitored. All frequencies are expressed in beats min^−1^.

The visual method (manual counts under a stereomicroscope) of measuring *f*_H_ was used to measure both short-term *f*_H_ and individual-chamber beat frequencies during the direct stimulus assays (see ‘Short-term *f*_H_ and SV beat frequency observations’, below).

The method used during the indirect stimulus assay to measure continuous *f*_H_ (see ‘Continuous *f*_H_ observations’, below) was performed using a cardiac monitoring device. This device allowed for hands-free recording of *f*_H_ over extended periods of time. At the time of recording, individual chambers could not be resolved; however, it did allow the detection of subtle changes in beat rate over time which was otherwise not apparent using the direct visualization method.

Throughout the paper, ‘baseline *f*_H_’ refers to pre-stimulus *f*_H_ measured by either short-term or continuous methods, depending on the assay.

#### Short-term *f*_H_ and SV beat frequency observations

Short-term *f*_H_ and SV beat frequency were measured in embryos at 60%, 70%, 80% and 90% of development using a Leica M80 stereomicroscope (Leica Microsystems, Deerfield, IL, USA). Each embryo was placed in a 50 µl droplet of isotonic ASW on a light pad, and temperature was continuously monitored with a Physitemp BAT-12 microprobe (Physitemp Instruments, Clifton, NJ, USA) and remained within 22–23°C.

Embryos rested undisturbed for 2 min before baseline recording. Beats were counted manually during two non-consecutive 30 s intervals separated by 30 s; values from both periods were doubled and averaged to determine beats min^−1^.

#### Continuous *f*_H_ observations

Continuous *f*_H_ was measured non-invasively in embryos at 60%, 70%, 80% and 90% of development using a fish embryo cardiology device (patent pending, Developmental Inc., Baton Rouge, LA, USA) that detects real-time cardiac signals from multiple individual embryos simultaneously. The device is a platform technology that consists of an array of chambers in a multi-well format, designed to hold and incubate embryos. In this on-chip design, fluidic controls enable chamber perfusion and remote orientation of the embryo to sensors integrated within the chamber. Sensors transduce heartbeats to voltage signals that depict a biphasic cardiac signal from each embryo, with clear systolic and diastolic peaks. Embryos were placed in the chambers with isotonic ASW and allowed to rest for 10 min before a baseline continuous *f*_H_ was recorded at room temperature (∼22–23°C), followed by measurement of responses to the indirect startle stimuli. Signals from the fish embryo cardiology device were output to a PowerLab PL3504 data acquisition and recording system (ADInstruments, Colorado Springs, CO, USA) and were analyzed and recorded at 1000 Hz with LabChart v8.1.28 software.

### Startle-induced atrial and ventricular arrest

To determine the developmental stage at which embryos first exhibit startle-induced atrial and ventricular arrest (indicative of vagal tone onset), the direct stimulus assay was performed at 40%, 50%, 60%, 70%, 80% and 90% of development.

A modified Armstrong (1931) method was used in which embryos were gently compressed under a thin, transparent acrylic plate (6×6×0.1 cm, 3.85 g) rather than a mica coverslip. The weight of the plate itself provided a controlled, sufficient compression; no additional manual pressure was required.

Embryos were oriented ventrally for clear visualization of the sinus venosus, atrium and ventricle, then compressed for 30 s. A positive startle response was defined as a complete, sustained (≥15 s) arrest of both atrium and ventricle. The same embryos were re-tested after 30 min exposure to 1 mmol l^−1^ atropine in isotonic ASW, and the cardiac startle response was re-examined.

### Startle-induced SV beat frequency changes

After establishing the developmental onset of vagally mediated atrial and ventricular arrest, SV beat frequency was used to assess the strength of parasympathetic influence. Because the SV continues beating during startle, reductions in SV beat frequency serve as an indirect measure of parasympathetic input. Embryos at 60%, 70%, 80% and 90% of development were first acclimated for 2 min in isotonic ASW at 22–23°C. Afterward, the SV beat frequency of undisturbed embryos, embryos immediately after startling and those 2 min post-startle were measured.

### Startle-induced bradycardia response

Responses to the indirect (vibrational) startle stimulus were measured in embryos at 60%, 70%, 80% and 90% of development using continuous *f*_H_ recordings obtained using the cardiac monitoring device (see ‘Continuous *f*_H_ observations’). Embryos were acclimated for 10 min in 22°C ASW before baseline recordings. The stimulus consisted of a single tap to the sensor chamber wall with a pen, creating a brief vibration. Five consecutive startle events were applied at 2 min intervals; post-startle *f*_H_ was measured 30 s before the next tap once baseline had recovered. Following the first trial, embryos were immersed in 1 mmol l^−1^ atropine in isotonic ASW for 30 min at 22°C and the assay repeated.

### Beat frequencies of individual heart chambers

Beat frequencies of the SV, atrium and ventricle were measured before and after the direct stimulus and again following atropine treatment at 60%, 70%, 80% and 90% of development. Beats were counted manually over 30 s under a stereomicroscope and doubled to obtain beats min^−1^.

### Conversion of developmental staging scale

To compare across studies using different staging schemes, the commonly used Armstrong and Child (1965) stages were converted to percentage of embryonic development (Table 1). This normalized embryos from both *F. grandis* and *F. heteroclitus* across environmental conditions and developmental durations, facilitating cross-study comparison.

**Table 1.** Alignment of embryonic developmental stages in Gulf killifish (*Fundulus grandis*) with the staging scale for Atlantic killifish (*Fundulus heteroclitus*)

	Embryonic development (%)						
	<40	40	60	70	80	90	100
Developmental stage	25–28	30	31	33	34	35–36	37–39
Approximate dpf (22°C)	3.5	8	9	10	11	12	≥12

Embryonic development is given as a percentage, where the hatching endpoint was at 100%. Developmental stage was as per Armstrong and Child (1965). dpf, days post-fertilization.

### Statistics

Short-term *f*_H_ was analyzed by Welch's ANOVA with Games–Howell *post hoc* tests for unequal group sizes. Binary (arrest/no-arrest) data were arcsine-transformed and evaluated by two-way repeated-measures ANOVA (factors: developmental stage, atropine treatment) with Holm–Šidák correction. Bradycardia data were analyzed by two-way repeated-measures ANOVA (factors: startle iteration, developmental stage). Individual-chamber beat frequencies were analyzed by three-way repeated-measures ANOVA (factors: drug treatment, chamber, developmental stage) with Bonferroni correction. Graphs were prepared in SigmaPlot 12. Results are means±s.e.m., and significance was accepted at *P*<0.05.

### Use of AI disclosure

No artificial intelligence (AI) tools were utilized in the preparation of this paper. All data collection, analysis and writing were conducted by the authors without the assistance of AI technologies.

## RESULTS

### Resting *f*_H_ throughout development

**Fig. 1.**
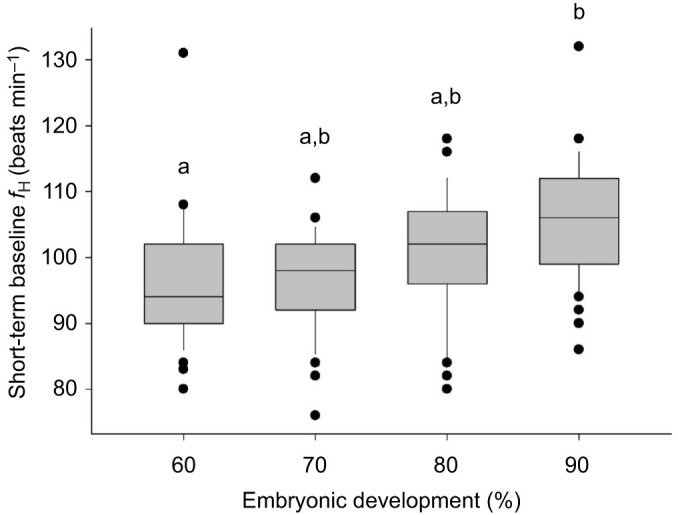
**Baseline embryonic heart rate during development in Gulf killifish (*Fundulus grandis*) embryos.** Short-term baseline heart rate (*f*_H_) measured visually under a stereomicroscope at 23°C at 60% (*n*=38), 70% (*n*=42), 80% (*n*=48) and 90% (*n*=48) of embryonic development (where the hatching endpoint was at 100%). Box plots show median, interquartile range (IQR) and whiskers extending to 1.5x IQR; individual data points are overlaid. Statistical significance (*P*<0.05) between groups is indicated by different lowercase letters.

Baseline short-term *f*_H_ increased from 96±1 beats min^−1^ at 60% to 106±1 beats min^−1^ at 90% of development (*P*<0.05), with no significant pairwise differences among intermediate stages (including 70% and 80%) (Fig. 1). Baseline continuous *f*_H_ likewise rose from 78±3 beats min^−1^ at 60% to 89±2 beats min^−1^ at 90% of development (see Table 2; Fig. S1).

**Table 2.** Reported heart rates of embryonic Gulf killifish (*F. grandis*) and Atlantic killifish (*F. heteroclitus*)

Species	Experimental treatment	*T* (°C)	Salinity (ppt)	pH	*f*_H_ (beats min^−1^) and developmental stage (%)				Ref.
Gulf killifish	Baseline (short-term)	23	10–12	7.8	96±2 (60%)	96±1 (70%)	100±1 (80%)	106±1 (90%)	1
	Baseline (continuous)	20	10–12	7.8	78±3 (60%)	83±5 (70%)	89±5 (80%)	89±2 (90%)	
	Salinity and pH	25	0.4	8	76 (∼30%)	89 (∼40%)	98 (70%)	105 (80%)	2^‡^
			7	8	71 (∼30%)	102 (∼40%)	107 (70%)	108 (80%)	
			15	8	67 (∼30%)	112 (∼40%)	94 (70%)	107 (80%)	
			30	8	78 (∼30%)	95 (∼40%)	99 (70%)	108 (80%)	
	Control	26.5	0	–	–			156±2 (100%)	3
	Crude oil vapor exposure				–			147±5 (100%)	
	Temperature	20	–	–	89 (∼30%)	121 (∼40%)	99 (70%)	118 (80%)	4^‡^
		23			87 (∼30%)	104 (∼40%)	120 (70%)	99 (80%)	
		26			98 (∼30%)	105 (∼40%)	121 (70%)	138 (80%)	
		30			97 (∼30%)	112 (∼40%)	108 (70%)	120 (80%)	
Atlantic killifish	Control	20±0.2	–	–	–			138 (≤90%)	5
	Vagal stimulation and recovery							113 (≤90%)	
	Control	20	5	–	118 (40%)	–			6*^,‡^
	1:5, 1:10, 1:20 PAH sediment exposure				106 (40%)	119 (40%)	128 (40%)	–	
	Control (PAH-sensitive), PAH-resistant, sensitive/resistant cross strain	25	15		121 (40%)	125 (40%)	132 (40%)	–	
					120 (80%)	131 (80%)	133 (80%)	–	
	Temperature	15	25	–	55 (∼40%)	–			7^‡^
		18			80 (∼40%)				
		21			110 (∼40%)				
		24			150 (∼40%)				
		27			180 (∼40%)				
		30			227 (∼40%)				
		33			255 (∼40%)				

Where applicable, values are presented as means±s.e.m.; dashes signify data were not reported. *T*, measured temperature. Original *F. heteroclitus* staging scale measured at 20±0.2°C (Armstrong and Child, 1965). *For assays performed at 25°C, *f*_^H^_ values represent means of subsets sampled from different polycyclic aromatic hydrocarbon (PAH) sediment sites. ^‡^In several references, mean *f*_^H^_ values were not reported numerically and were instead estimated from published graphical data. References: ^1^present study; ^2^Brown et al., 2012; ^3^Gurung et al., 2021; ^4^Brown and Green, 2014; ^5^Armstrong, 1931; ^6^Bozinovic et al., 2022; ^7^Blanchard et al., 2024.

### Effect of startling on SV beat frequency

**Fig. 2.**
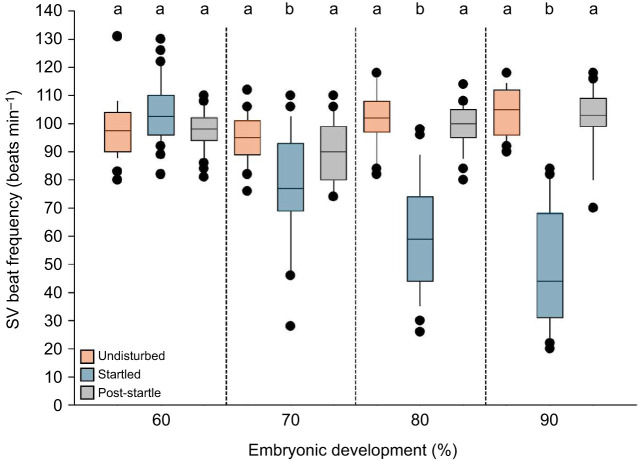
**Sinus venosus beat frequency in response to the direct startle stimulus.** Sinus venosus (SV) beat frequency of embryos at 60% (*n*=26), 70% (*n*=26), 80% (*n*=24) and 90% (*n*=24) of development measured (i) while undisturbed for 2 min, (ii) immediately following 30 s of startle stimulus (compression of the embryo) and (iii) after 2 min recovery (post-startle). Box plots show median, interquartile range (IQR) and whiskers extending to 1.5x IQR; individual data points are overlaid. Statistical significance (*P*<0.05) is denoted by different lowercase letters.

At 60%, the direct, physical startle stimulus did not change SV beat frequency (*P*>0.05). Beginning at 70% and continuing through 80% and 90%, startle significantly reduced SV beat frequency relative to both 2 min undisturbed (pre-startle) and 2 min post-startle values (*P*<0.05), after which beat frequency recovered (Fig. 2). The magnitude of this startle-induced reduction increased from 70% onward. Across stages, undisturbed and post-startle SV rates did not differ significantly (*P*>0.05).

### Developmental onset of transient atrial and ventricular arrest response

**Fig. 3.**
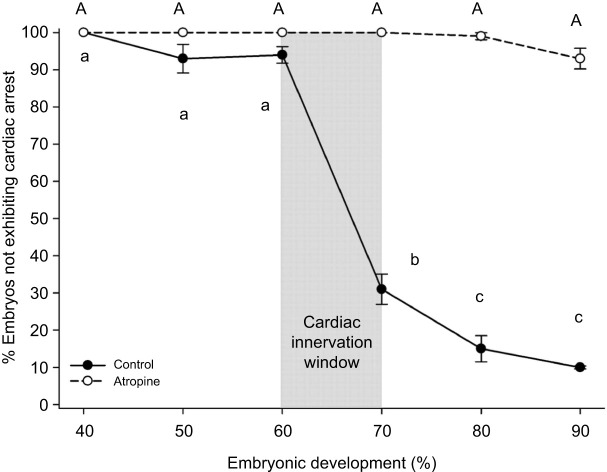
**Developmental onset of startle-induced atrial and ventricular arrest in Gulf killifish embryos.** Percentage of embryos failing to undergo complete atrial and ventricular arrest following direct startle stimulation at 40% (*n*=33), 50% (*n*=44), 60% (*n*=124), 70% (*n*=130), 80% (*n*=105) and 90% (*n*=85) of development. Both pre-atropine control (solid line) and post-atropine (dashed line) values are shown as means±s.e.m. Statistical differences (*P*<0.05) are indicated by distinct lettering; lowercase, pre-atropine; uppercase, post-atropine. In some cases, error bars are smaller than the symbols themselves.

At ≤60% of development, only 6% (8 of 124) of embryos showed transient atrial and ventricular arrest during the direct stimulus assay (Fig. 3). By 70%, ∼70% (90 of 130) of embryos exhibited arrest, significantly higher (*P*<0.001) than ≤60% embryos. The percentage of embryos displaying arrest continued to rise at 80% and through 90% to hatch. Atropine treatment abolished the startle-induced arrest response in almost all ≥60% embryos (*P*<0.001).

### Development of transient bradycardia response

**Fig. 4.**
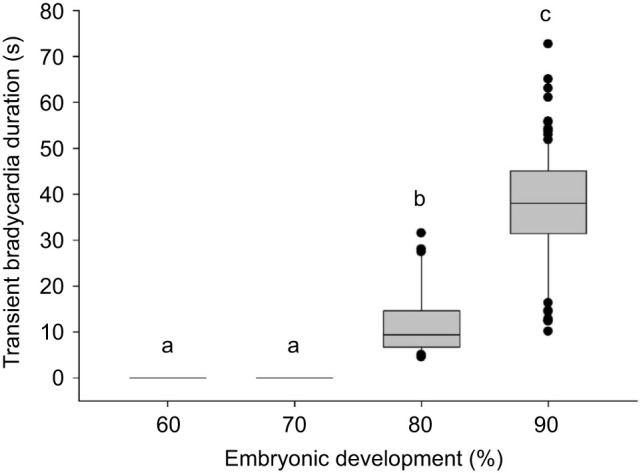
**Transient bradycardia induced by the indirect (vibrational) cardiac startle stimulus.** Duration of bradycardia following indirect startle stimuli at 60% (*n*=11), 70% (*n*=11), 80% (*n*=5) and 90% (*n*=21) of development, derived from continuous *f*_H_ recordings (fish embryo cardiology device). Box plots show median, interquartile range (IQR) and whiskers extending to 1.5x IQR; individual data points are overlaid. Statistical significance (*P*<0.05) is denoted by different lowercase letters.

**Fig. 5.**
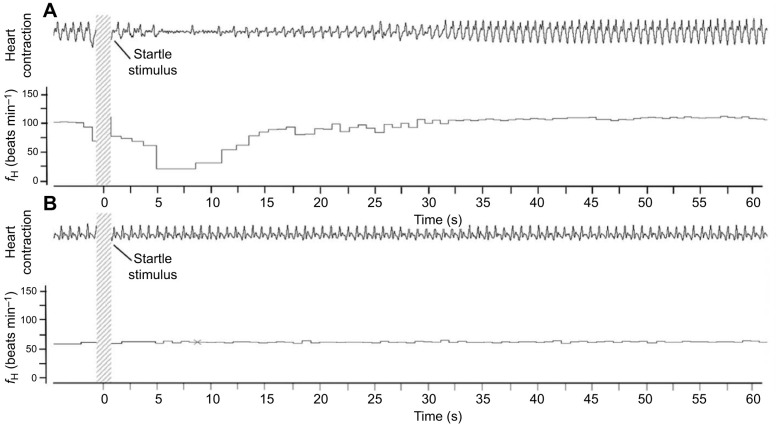
**Representative continuous *f*_H_ traces showing the cardiac startle response to indirect (vibrational) stimulation.** (A) Pre-atropine (control) trace illustrating transient bradycardia following a single chamber-tap stimulus. (B) Post-atropine trace showing elimination of the bradycardic response.

No habituation to repeated indirect stimuli was detected at any measured stage (Fig. 4), and the indirect (vibrational) stimulus did not alter continuous *f*_H_ at 60% or 70% of embryonic development. Prior to atropine, after each startle event, *f*_H_ returned to pre-startle values. Atropine abolished the bradycardia response at all stages tested (Fig. 5).

### Beat frequencies of specific heart chambers

#### Sinus venosus

**Fig. 6.**
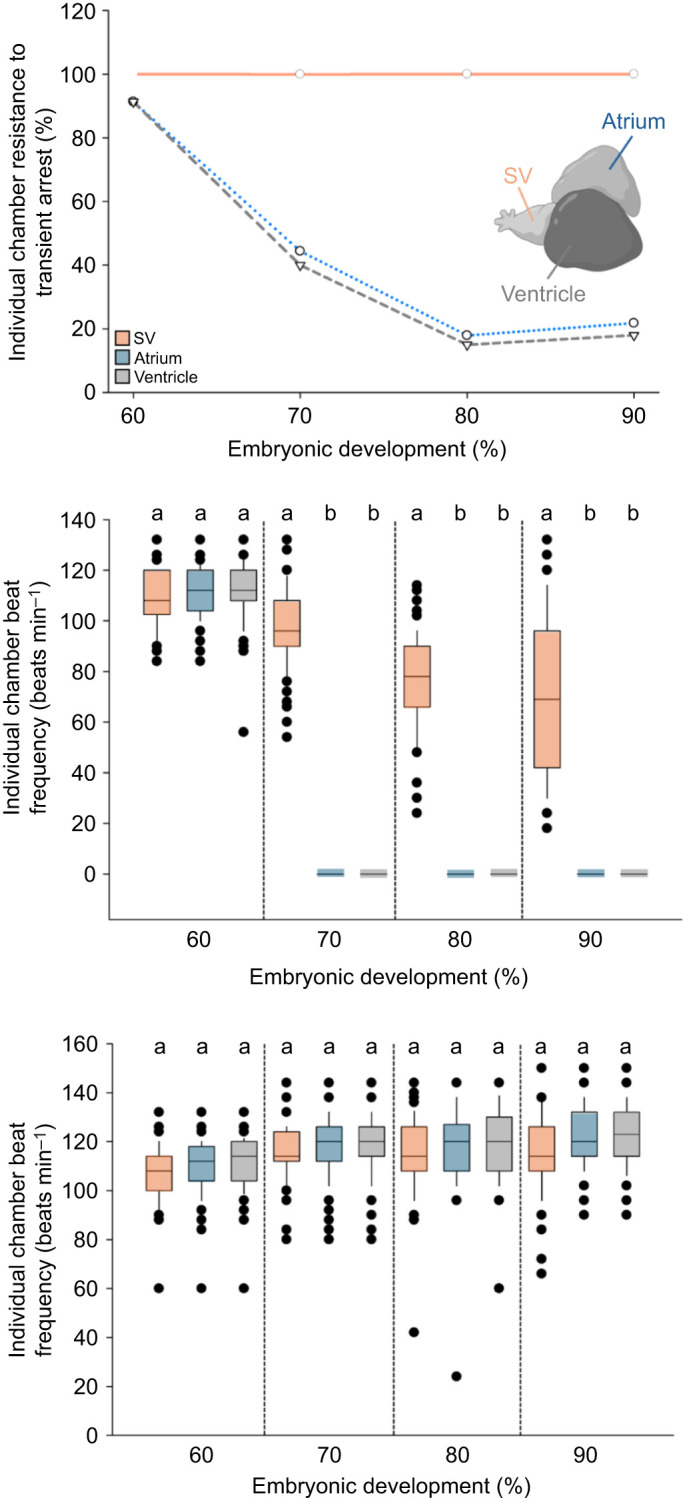
**Chamber-specific cardiac responses to direct startle stimulation before and after atropine treatment.** (A) Percentage of pre-atropine embryos lacking arrest of the SV, atrium and ventricle after direct stimulation (gentle compression) at 60% (*n*=92), 70% (*n*=95), 80% (*n*=84) and 90% (*n*=72) of development. At 70%, 80% and 90% of development, datapoints for the atrium are offset from the corresponding ventricle datapoints by ±2% to allow unobstructed visualization of both lines. (B,C) Mean beat frequency (beats min^−1^) of individual heart chambers pre- (B) and post-atropine (C). Box plots show median, interquartile range (IQR) and whiskers extending to 1.5x IQR; individual data points are overlaid. Statistical significance (*P*<0.05) is denoted by different lowercase letters.

From 70% onward, the direct startle stimulus produced transient arrest of the atrium and ventricle while the SV continued to beat (Fig. 6A).

SV beat frequency declined modestly as development progressed (day-to-day) in undisturbed embryos (‘floor effect’) and was further depressed during startle. Cumulatively, direct startle produced an ∼35% decrease across 60–90% of embryonic development (*P*<0.001) (Fig. 6B). At 60%, atropine had no effect on SV rate; at 70–90%, atropine eliminated the startle-related depression and significantly increased SV beat frequency (*P*<0.001) (Fig. 6C).

#### Atrium and ventricle

At 60%, ∼91% of embryos maintained regular atrial and ventricular beating during startle. At 70%, only 39% maintained atrial and ventricular beating; most exhibited arrest. Arrest prevalence increased further at 80% and 90%, with only 18% and 25% unaffected, respectively. The proportion with atrial and ventricular arrest was therefore significantly greater at 70–90% than at 60% (*P*<0.001) (Fig. 6A,B). Atropine abolished the atrial and ventricular arrest response at 70–90% and significantly increased atrial and ventricular beat frequencies (*P*<0.001), while having no effect at 60% (*P*>0.05) (Fig. 6C). Across all stages and treatments, atrial and ventricular beat frequencies maintained a 1:1 beat ratio for each heartbeat (no significant differences between chambers).

## DISCUSSION

### Developmental trajectory of embryonic *f*_H_

The rise in *f*_H_ in *F. grandis* embryos followed the general vertebrate paradigm of progressively increasing *f*_H_ during development (Burggren et al., 2017), as significant increases in short-term *f*_H_ were observed between 60% and 90% of development (Fig. 1). Although these measurements were collected at discrete developmental stages, the data illustrate the trend of rising *f*_H_ as embryos approached hatching.

This gradual increase in *f*_H_ likely reflects the normal maturation of intrinsic cardiac mechanisms common to developing teleosts. As the heart forms and its structures differentiate, the excitability of the sinoatrial region increases, cardiomyocytes proliferate and electrical conduction between chambers becomes more coordinated (Baillie et al., 2023; Burggren et al., 2017; Leone et al., 2015; Stoyek et al., 2022). Collectively, these developmental changes strengthen pacemaker function and enhance overall cardiac performance during development.

To contextualize our findings, we compared our *f*_H_ data with previous studies reporting embryonic *f*_H_ in *Fundulus* species (Table 2). Based on these earlier reports, our results fall within the normal range observed across different *Fundulus* populations and laboratory conditions. Although absolute *f*_H_ values can vary with temperature, salinity and experimental setup, the trajectory of developmental *f*_H_ in our embryos closely matches the overall trend reported within these comparison studies. These baseline data from our study provide a reference for future investigations into the onset and progression of parasympathetic modulation during embryogenesis.

### Onset of parasympathetic cardiac regulation

In teleost fishes, cardiac rhythm originates from autorhythmic cardiomyocytes within the sinoatrial region, located at the junction between the sinus venosus and atrium. These pacemaker cells fulfill a role analogous to the mammalian sinoatrial node, despite teleosts lacking a discrete His–Purkinje conduction system (Gauvrit et al., 2022; Poon and Brand, 2013). Spontaneous depolarization in the sinoatrial region generates electrical impulses that sequentially propagate through the single atrium and ventricle, initiating the systolic phase of the cardiac cycle (Burggren et al., 2017; Jensen et al., 2014; Tessadori et al., 2012).

Parasympathetic modulation of this rhythm begins once the vagus nerve functionally innervates the sinoatrial region. Here, acetylcholine released from post-ganglionic fibers binds to muscarinic M_2_ receptors, slowing diastolic depolarization and thereby reducing *f*_H_ (Hsieh and Liao, 2002; Taylor et al., 2009). The onset of this inhibitory influence marks the emergence of vagal tone during development, enabling the embryonic heart to dynamically respond to physiological and environmental stimuli.

Having characterized the developmental trajectory of intrinsic cardiac function, our data indicate that *F. grandis* embryos, like their Atlantic counterparts, acquire functional vagal regulation well before hatching, establishing parasympathetic control of cardiac rhythm *in ovo*. Once this tone is established, involuntary cardiac responses can be elicited by both direct and indirect startle stimuli.

**Fig. 7.**
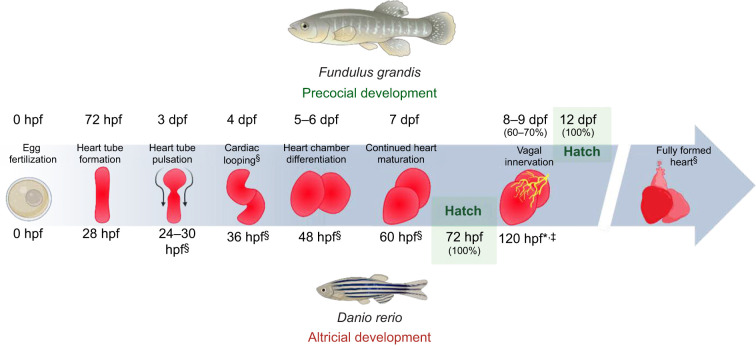
**Comparative developmental timelines of heart formation and cardiac vagal innervation in the precocial Gulf killifish (*F. grandis*) and altricial zebrafish (*Danio rerio*).** Contrasting timing of major cardiac milestones between *F. grandis* and *D. rerio*. The developmental window of vagal innervation is shown, occurring before hatching in Gulf killifish and at or shortly after hatching in zebrafish. hpf, hours post-fertilization; dpf, days post-fertilization. *Schwerte et al., 2006; ^§^Bakkers, 2011; ^‡^Mann et al., 2010. Created in BioRender by Williams, S., 2026. https://BioRender.com/aehnojr. This figure was sublicensed under CC-BY 4.0 terms.

This precocial onset of vagal tone contrasts with the delayed parasympathetic development observed in more altricial teleosts such as zebrafish, in which functional vagal innervation occurs only after hatching (Mann et al., 2010; Schwerte et al., 2006). The comparative timeline in Fig. 7 illustrates these developmental differences, highlighting how extended embryogenesis in *F. grandis* allows the early establishment of functional autonomic control relative to faster-developing altricial species. This comparison provides a useful visual context for understanding the onset and functional outcomes of parasympathetic regulation described in the following sections.

### Cardiac startle response phenotypes

Having identified the onset of functional parasympathetic control, we next examined how this vagal influence manifests during exposure to startle stimuli. During our characterization of cardiac startle responses in Gulf killifish embryos, two distinct, vagally mediated cardiac response phenotypes were identified: (1) a transient (≥15 s) yet complete arrest of both the atrium and ventricle, and (2) a transient (≤1 min) bradycardia without full arrest. The exhibition of either phenotype depended on both the developmental age of the embryos and the method of the applied cardiac startle stimulus (i.e. direct versus indirect stimulation).

#### Transient atrial and ventricular arrest response

An insignificant number of Gulf killifish embryos exhibited startle-induced arrest at 60% of development. However, upon reaching 70% of developmental, a significant (*P*<0.001) number of embryos experienced transient atrial and ventricular arrest when startled. This suggests that functional vagal innervation of the heart occurs in a window shortly before reaching 70% of development (Fig. 3). Administration of atropine abolished this response, confirming its parasympathetic origin. By 90% of development, nearly all embryos displayed a transient arrest of the atrium and ventricle following direct mechanical stimulation (gentle compression).

The strong susceptibility to arrest near 90% of development, which corresponds with typical hatching readiness, suggests that vagal maturation coincides with the transition to *ex ovo* life. Shifts in the timing or strength of this vagal innervation could therefore serve as diagnostic indicators of abnormal embryonic development.

Few studies have examined startle-induced atrial and ventricular arrest in teleost embryos; however, transient cardiac arrest has been documented in adult fishes exposed to acute fright stress (Barham et al., 1985) or intense visual stimuli (Laming, 1987). These parallels suggest that the killifish embryonic cardiac startle response may represent the developmental precursor to the adult autonomic reflex to startling stimuli, linking early developmental physiology with mature cardiac regulation in these species.

#### Transient bradycardia response

The indirect startle stimulus elicited a distinct cardiac response phenotype – an intense but reversible bradycardia lasting ≤1 min (Fig. 5). This response, detected through high-resolution beat-period analysis using the cardiac monitoring device, was abolished by atropine, confirming vagal mediation. Interestingly, this transient bradycardia response first appeared at 80% of development, later than the transient arrest response, which emerged at 70% following the stronger direct startle stimulus (Fig. 4). This suggests that cardiac responsiveness to more subtle stimulation emerges only during late-stage embryonic development as vagal tone matures. In contrast to the transient arrest response produced by the stronger direct stimulus, this bradycardic response represents an alternative startle phenotype that allows rapid recovery to pre-startle cardiac performance.

#### Habituation to the indirect cardiac startle stimulus

Although the duration of transient bradycardia increased as development progressed, evidence of a maturing parasympathetic response, no habituation to repeated startle events was observed in Gulf killifish embryos. Habituation to the stimulus would have indicated a third cardiac startle response phenotype, integrating an anticipatory behavioral component with the involuntary physiological reaction of the heart (Kepler et al., 2020).

To test for habituation, embryos at 60%, 70%, 80% and 90% of development were each subjected to five successive indirect startle stimuli (Fig. 4). By 90% of development, embryos exhibited longer bradycardia recovery times than at 80%, but recovery duration remained consistent across startle events within each group. In contrast, Mann et al. (2010) reported habituation in zebrafish larvae, where repeated electrical shocks produced progressively attenuated bradycardia responses. Although behavioral habituation has been documented in many adult teleost species (Laming, 1987), similar cardiac habituation has not been described in teleost embryos to our knowledge.

### Mechanistic basis of response phenotypes

To better understand the physiological basis of the arrest and bradycardia phenotypes described above, we examined the beating patterns of individual heart chambers in embryonic Gulf killifish (Fig. 6A). Stimulation of the vagus nerve appears to induce an intermittent, atropine-sensitive conduction block, resulting in transient arrest of both the atrium and ventricle while the SV continues beating, albeit at a reduced rate. To explore this further, we analyzed the beat characteristics of individual heart chambers both before (pre-atropine) and after (post-atropine) parasympathetic blockade.

#### SV beat characteristics

The structure and cardiomyocyte composition of the SV vary among teleost fishes (Farrell and Jones, 1992). The SV contains autorhythmic cardiomyocytes capable of initiating contractions largely independent of autonomic input. As such, it is unsurprising that SV beating persisted despite applying the direct startle stimulus (compression of the embryo).

We observed that startle stimulation reduced SV beat frequency beginning at 70% of development, though the rate gradually recovered following stimulation (Fig. 2). Across successive developmental stages, mean SV beat frequency also declined slightly prior to atropine treatment (‘floor effect’), consistent with the natural slowing of intrinsic pacemaking as the heart matures rather than residual effects from earlier startle trials (Barrionuevo and Burggren, 1999; Holeton, 1971) (Fig. 6B). After atropine administration, SV beat frequency increased significantly (Fig. 6C). Therefore, the uninterrupted SV beating during startling, coupled with the post-atropine increase in SV beat frequency, indicates that the pacemaker region is influenced – but not wholly governed – by parasympathetic input (Le Mével et al., 2012; Shiels, 2017).

Armstrong (1931) similarly observed a slowing of ‘heart rate’ in 9 dpf (60–70% development) Atlantic killifish embryos following startling, which he attributed to vagal inhibition. Based on our observations that atrial and ventricular arrest occur at this stage while SV beating persists, it is likely that Armstrong's post-startle measurements reflected SV beat frequency rather than complete cardiac activity, as the atrium and ventricle may have remained temporarily arrested. His observation that this arrest could persist for 3–5 min further supports this interpretation. Collectively, both Armstrong's (1931) observations and our results indicate that SV activity is subject to parasympathetic modulation – exhibiting a negative chronotropic effect – during the later stages (≥70%) of embryonic development.

#### Atrial and ventricular beat characteristics

Before 70% of development, the atrium and ventricle exhibited no response to startle stimulation, consistent with the absence of functional vagal tone (Fig. 6B). However, at 70% and beyond, both chambers exhibited complete arrest upon startling, while the SV continued to beat. Following atropine treatment, beat frequencies of both chambers increased significantly, indicating removal of parasympathetic inhibition (Fig. 6C). Together, these findings indicate that functional vagal tone mediates a reversible, atropine-sensitive suppression of atrial and ventricular activity during startle. This suggests that a vagally mediated conduction block is responsible for the transient arrest of the atrium and ventricle rather than disruption of intrinsic pacemaking.

### Significance of precocial cardiac regulation

In most vertebrates, parasympathetically mediated cardiac startle responses do not appear until after hatching or birth. Consequently, studies of developing cardiac regulation have largely focused on altricial animals. Across vertebrate classes, parasympathetic-mediated bradycardia has been documented as part of the acoustic startle response in humans, porpoises and rodents exposed to air-puff stimuli (Battaglia et al., 2024; Casto and Printz, 1990; Chen et al., 2014; Elmegaard et al., 2021), and in the response to hypoxia in fish and fetal sheep (Cohn et al., 1980; Giussani et al., 1993; Joyce and Wang, 2022). Methodologically, the closest comparison to the present study comes from zebrafish, where newly hatched larvae exhibit a startle-induced bradycardia lasting ∼2 s after brief, intermittent electrical shocks (Mann et al., 2010). Mann et al. (2010) also showed that zebrafish larvae can habituate to repeated startle events, with bradycardic responses progressively attenuating over time. Together, these observations highlight both the evolutionary conservation and adaptive value of parasympathetic cardiac control across vertebrates.

However, killifish diverge from this general paradigm of autonomic nervous system development, as both Gulf (*F. grandis*) and Atlantic (*F. heteroclitus*) species exhibit the precocial onset of vagal tone well before hatching, coinciding with their comparatively long embryonic periods (10–14 days). While environmental factors can modulate *f*_H_ in *Fundulus* embryos, other killifish taxa display even more extreme developmental adaptations. For instance, the annual killifish (*Austrofundulus limnaeus*) can enter diapause, suspending development until environmental conditions become favorable for hatching (Gao et al., 2022; Martin and Podrabsky, 2017; Podrabsky et al., 2010). During diapause, oxygen consumption is reduced by up to 90%, with *f*_H_ dropping below 30 beats min^−1^ (Podrabsky and Hand, 1999).

Although Gulf killifish lack a true diapause stage, to a limited degree hatching can be delayed under adverse environmental conditions such as fluctuating salinity, temperature, pollution or tidal cycles (Brown and Green, 2014; Brown et al., 2011; Brown et al., 2012; Dubansky et al., 2013; Greeley and MacGregor, 1983; Singh et al., 2024; Walsh and Roden, 2024). In these circumstances, the early establishment of parasympathetic cardiac regulation may therefore confer an adaptive advantage, allowing embryos to involuntarily modulate cardiac output and thereby reduce energy expenditure during unpredictable environmental events.

Finally, early maturation of vagal tone may also function as a physiological safeguard, improving tolerance to developmental stressors and enhancing embryo survival in variable estuarine habitats (Alonso et al., 2024; Brennan et al., 2016; Earhart et al., 2024; Oziolor et al., 2019; Whitehead et al., 2017). In this sense, *Fundulus* embryos provide a unique model for understanding how autonomic control of the heart enhances resilience during early life stages, bridging the gap between developmental physiology and environmental adaptation.

### Conclusion

Building on Armstrong's (1931) original observations in Atlantic killifish embryos, our findings in the closely related Gulf killifish confirm that parasympathetic cardiac regulation emerges well before hatching. This early vagal innervation represents a clear example of an exception to the vertebrate paradigm in which functional vagal tone typically develops only near or after hatching or birth, with few known exceptions. The ability of *F. grandis* embryos to exhibit distinct, atropine-sensitive cardiac startle responses *in ovo* suggests that autonomic control of *f*_H_ is functionally established prior to hatching. This precocial autonomic control may enhance embryonic resilience and hatching flexibility under fluctuating environmental conditions.

More broadly, these results position *Fundulus* embryos as tractable vertebrate models for studying the developmental origins of autonomic cardiac regulation, providing a benchmark for comparative analyses across vertebrate taxa and furthering our understanding of how developing organisms coordinate physiological and environmental influences during early life.

## Supplementary Material

10.1242/jexbio.251772_sup1 Supplementary information
