# Supplementary material for: Precocial parasympathetic-mediated cardiac regulation in embryonic gulf killifish (Fundulus grandis)
Source: J Exp Biol. 2026 Apr 30;229(9):jeb251772. doi: 10.1242/jeb.251772 (PMC13200724; doi:10.1242/jeb.251772)
Supplement: Supplementary information [file jexbio-229-251772-s1.pdf]

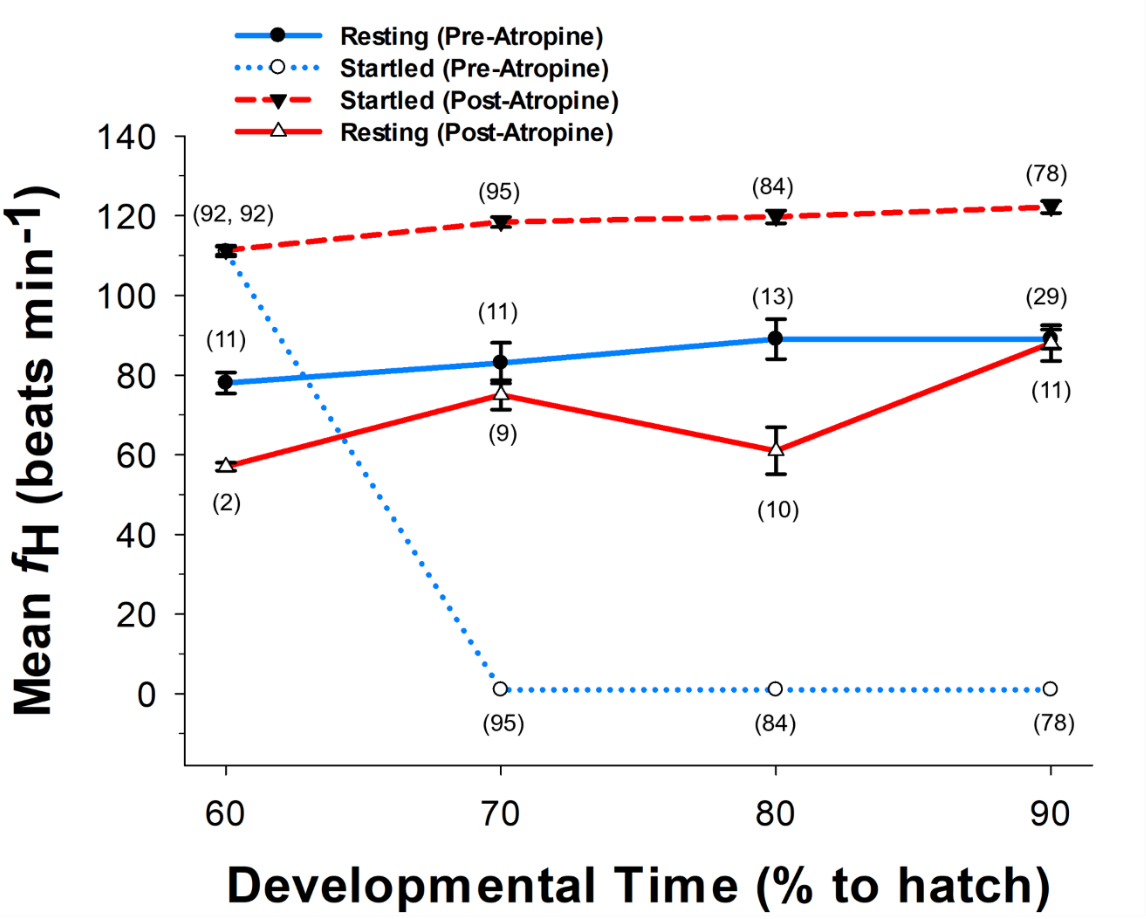


**Fig. S1. Mean $f_H$ during resting and startled states before and after atropine treatment.** Mean $f_H$ (beats·min$^{-1}$) measured at 60%, 70%, 80%, and 90% of embryonic development under resting and startled conditions before and after atropine treatment. Dashed lines represent short-term $f_H$ measurements from embryos subjected to a direct startle stimulus (manual compression), where $f_H$ was calculated manually by direct observation under a stereomicroscope. Solid lines represent continuous $f_H$ measurements from embryos subjected to an indirect startle stimulus (vibrational), recorded passively using the fish embryo cardiology device. Data are presented as mean ± s.e.m. Numbers in parentheses indicate sample sizes for each developmental time point and treatment condition.
